# Supplementary material for: Long-Term Outcome of Leptospirosis Infection with Acute Kidney Injury
Source: Biomedicines. 2022 Sep 20;10(10):2338. doi: 10.3390/biomedicines10102338 (PMC9598535; doi:10.3390/biomedicines10102338)
Supplement: Supplementary file 1 [file biomedicines-10-02338-s001.zip › biomedicines-1890319-supplementary.pdf]

Table S1. ICD-9-CM code of comorbidities.

**Comorbidities**	**ICD-9-CM Codes**
Diabetes mellitus	250
Hypertension	401-405
Hyperlipidemia	272.4
Gout	274
Chronic hepatitis	070.32, 070.54
Peripheral arterial occlusive disease	443.9
Coronary artery disease	410-414
Heart failure	428
Stroke	430-438
